# Variant Branching Pattern of Axillary Artery: A Case Report

**DOI:** 10.1155/2012/976968

**Published:** 2012-10-09

**Authors:** Swamy Ravindra Shantakumar, K. G. Mohandas Rao

**Affiliations:** Department of Anatomy, Melaka Manipal Medical College (Manipal Campus), Manipal University, Karnataka, Manipal 576 104, India

## Abstract

During routine dissection of an approximately 50-year-old male cadaver for the undergraduate medical students at Melaka Manipal Medical College, Manipal University, Manipal, we came across a variation in branching pattern of right axillary artery. The second part of axillary artery gave rise to a common trunk which divided into the subscapular and lateral thoracic arteries. The third part of right axillary artery gave rise to anterior and posterior circumflex humeral arteries. Variations in the branching pattern of axillary artery are important for the surgeons performing interventional or diagnostic procedures in cardiovascular diseases.

## 1. Introduction

**Figure 1:**
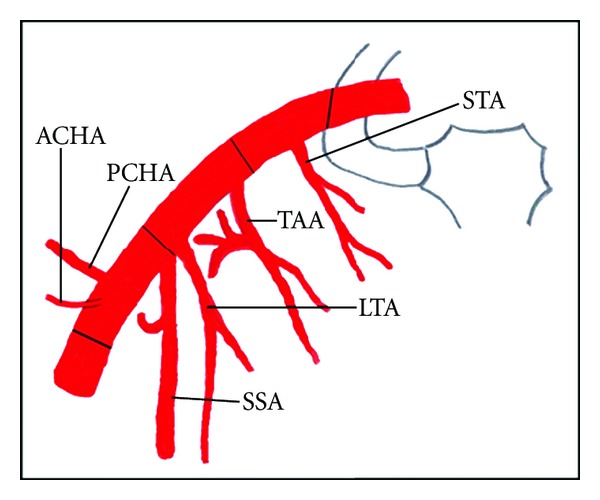
Schematic representation of normal branching pattern of axillary artery showing superior thoracic artery (STA) from the first part; thoracoacromial artery (TAA) and lateral thoracic artery (LTA) from the second part; subscapular artery (SSA), anterior circumflex humeral artery (ACHA), and posterior circumflex humeral artery (PCHA) from the third part of axillary artery.

Axillary artery is a continuation of subclavian artery, extending from the outer border of first rib to the lower border of teres major muscle where it continues as brachial artery. The pectoralis minor muscle is related anteriorly to the axillary artery and it divides axillary artery into three parts, first part extending from outer border of first rib to the upper border of pectoralis minor muscle, second part lies behind the pectoralis minor muscle, and third part extending from lower border of pectoralis minor muscle to lower border of teres major muscle. Axillary artery normally gives off superior thoracic artery from the first part, lateral thoracic artery and thoracoacromial artery from the second part, and subscapular, anterior circumflex humeral and posterior circumflex humeral arteries from the third part (Figure 1). Subscapular artery divides into circumflex scapular and thoracodorsal arteries [1]. 

## 2. Case Report

**Figure 2:**
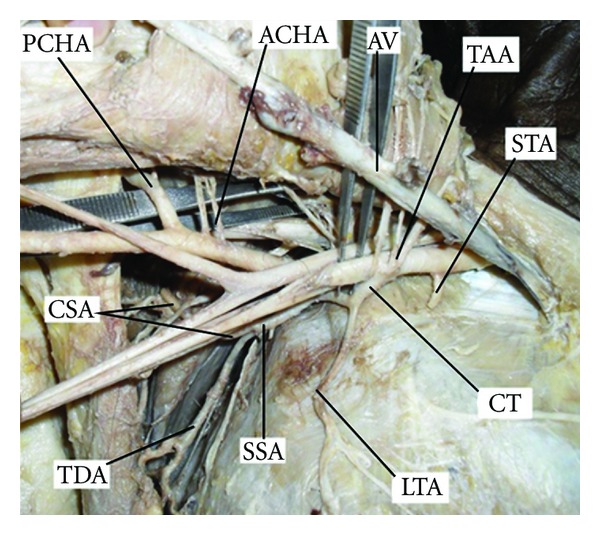
Dissection of right axilla shows the variant branching pattern of axillary artery. (AV: axillary vein, ACHA: anterior circumflex humeral artery, CT: common trunk, CSA: circumflex scapular artery, LTA: lateral thoracic artery, PCHA: posterior circumflex humeral artery, SSA: subscapular artery, STA: superior thoracic artery, TDA: thoracodorsal artery, and TAA: thoracoacromial artery).

During routine dissection for undergraduate Medical students of Melaka Manipal Medical College, Manipal University, Manipal, we came across a variation in branching pattern of right axillary artery in a male cadaver of approximately 50 years of age. The left axillary artery was found to be normal. The first part of axillary artery gave rise to superior thoracic artery as usual, but the second part gave a common trunk which divided into the lateral thoracic and subscapular arteries. The third part of the artery gave origin to anterior and posterior circumflex humeral arteries (Figure 2). This is a novel observation and has not been described earlier on radiological studies. The subclavian artery and brachial artery of both the right and left sides had normal branching pattern.

## 3. Discussion

Variation of branching pattern of axillary artery is common. Samta et al. reported that 28% of cases studied have variation in branching pattern of axillary artery. Subscapular artery has been found to be arising from 2nd part of axillary artery in 4% cases and in up to 30% of it arises from a common trunk with posterior circumflex humeral artery [2]. Huelke in his study reported that subscapular artery arises from the first part of axillary artery in 0.6% cases, from the second part in 15.7% cases, and from the third part in 79.2% cases [3]. Variation in branching pattern of axillary artery has been reported earlier by Rao et al., in which the third part of the left axillary artery gave origin to subscapular, anterior and posterior circumflex humeral, profunda brachii, and ulnar collateral arteries from a common trunk [4]. Samuel et al. reported about a common trunk from the third part of the axillary artery giving rise to anterior circumflex humeral, posterior circumflex humeral, and subscapular arteries and which then descended into the arm to give radial collateral, middle collateral and continued as the superior ulnar collateral artery [5]. In another case report, Srimathi reported that a common trunk from the second part of axillary artery gave origin to thoracoacromial, lateral thoracic, subscapular, and posterior circumflex humeral arteries [6]. George et al. reported a case in which the axillary artery gave a large abnormal arterial trunk which in turn divided into a common circumflex humeral-subscapular trunk and profunda brachii artery [7]. In the present case the second part of the axillary artery gave rise to common trunk which divided into lateral thoracic and subscapular arteries. Variation in branching pattern of axillary artery may be due to the defects in embryonic vascular network occurred at any stage by the arrest of development. The developmental defects of surrounding tissue may also lead to vascular variations [8].

Variations in branching pattern of axillary artery should be kept in mind while performing bypass between the axillary and subclavian artery in surgical treatment of subclavian artery occlusion [8]. The common trunk as in the present case can be used for bypass. Aneurysm and trauma of axillary artery may require reconstructive operation and variations as in the present case may present difficulties in the procedure. Aneurysms of the axillary artery and its branches may appear in baseball pitchers [9]. Repetitive positional compression of the axillary artery in athletes can cause focal intimal hyperplasia, aneurysm formation, segmental dissection, and branch vessel aneurysms. These conditions favour thrombosis and distal embolism and may need positional arteriography for diagnosis [10]. Variant branches of axillary artery as in the present case are also prone for such conditions. The axillary arteries have been successfully used as the cannulation site in cardiopulmonary bypass, thoracic, and aortic procedures, for insertion of intra-aortic balloon pumps and it is under consideration for use as an inflow vessel in coronary artery surgery [11]. Variant common trunks from axillary artery can be considered for cannulation. Radiological studies can thus be performed before proceeding to the above mentioned procedures. All these applications make present variation noteworthy.
